# Application of electrogastrogram in assessment of gastric motility in acute pancreatitis

**DOI:** 10.3389/fphys.2023.1281342

**Published:** 2023-11-13

**Authors:** Ying Cai, Jinyun Wang, Deqiang Huang, Lingyu Luo

**Affiliations:** ^1^ Department of Gastroenterology, The First Affiliated Hospital of Nanchang University, Nanchang, Jiangxi, China; ^2^ Department of Gastroenterology, Gaoxin Branch, The First Affiliated Hospital of Nanchang University, Nanchang, Jiangxi, China

**Keywords:** acute pancreatitis, electrogastrogram, gastric motility, severity, percentage of gastric bradycardia

## Abstract

**Background:** Electrogastrogram (EGG) can reflect gastric motility disorders in many diseases, but its application in acute pancreatitis (AP) has not been studied. Therefore, our study aimed to investigate the value of EGG in assessing the existence of gastric motility disorder in patients with AP and in predicting the severity of AP.

**Methods:** Patients with AP admitted to the First Affiliated Hospital of Nanchang University from June 2020 to December 2020 were enrolled. Five EGG parameters (Percentage of normal gastric slow wave (PNGSW), main frequency, average frequency, percentage of gastric tachycardia (PGT), percentage of gastric bradycardia (PGB)) were collected. The receiver operating characteristic (ROC) curve was constructed to judge the predictive value of EGG parameters to AP severity.

**Results:** The PNGSW in AP patients was significantly lower than that of the control group (*p* < 0.05), and the PGB was higher in AP patients than that of the control group (*p* < 0.05). The area under curve (AUC) of the PNGSW and the PGB in diagnosing non-mild acute pancreatitis (N-MAP) were 0.777 (95% CI: 0.676-0.877, *p* < 0.001) and 0.775 (95% CI: 0.670-0.879, *p* < 0.001) respectively. After combining with C-reactive protein, the accuracy, sensitivity and specificity of predicting N-MAP were improved.

**Conclusion:** EGG parameters can well reflect the gastric motility disorder of AP patients. The PNGSW and the PGB can be used to predict the occurrence of N-MAP.

## 1 Introduction

Acute pancreatitis (AP) is one of the most common diseases of the gastrointestinal tract and a rapidly developing inflammatory process of the pancreas (Zhou et al., 2019). In recent years, with the increasing incidence and hospitalization rate of AP, the cost of hospitalization has also increased. In the United States, the annual cost of AP is as high as $ 2.6 billion, which places a heavy burden on medical resources and social economy (Lankisch et al., 2015; Krishna et al., 2017; Peery et al., 2018). Most AP patients have a mild condition and a good prognosis, but there are still 15%–20% of patients who will develop into severe acute pancreatitis (SAP) with a mortality rate of up to 30% (Waller et al., 2018; Zhou et al., 2019). Therefore, early prediction of the severity of AP is of great significance for reducing mortality and improving prognosis.

Patients with AP usually have gastrointestinal motility disorders (Wang et al., 2003; Seerden et al., 2005). Studies have shown that in AP patients, especially SAP, the degree of gastrointestinal motility disorder is closely related to the severity of the disease, which is an important cause of aggravation and even death (Adike and Quigley, 2014; Wu et al., 2017). Therefore, early assessment of the presence of gastrointestinal motility disorders in AP patients is of great significance for clinical guidance of follow-up treatment. In clinical practice, gastric emptying detection is usually needed to identify the presence of gastric motility disorders. At present, there were four main methods for evaluating gastric motility disorders (delayed gastric emptying): radionuclide imaging (gastric emptying scintigraphy), 13C gastric emptying expiratory test (13C-GEBT), wireless power capsule (WMC) and gastroparesis basic symptom index (GCSI). The above methods are difficult to carry out in clinical practice due to high radioactivity, high cost and poor objectivity (Keller et al., 2018). Therefore, it is urgent to find an objective, simple and clinically easy method to evaluate the presence of gastric motility disorders in AP patients.

Electrogastrogram (EGG) is a non-invasive examination that monitors the electrical activity of gastric smooth muscle by placing electrodes at a specific surface position on the abdomen. Because of its non-invasive, painless, simple and objective characteristics, it has gradually become a new way to detect gastric motility in clinical practice (Gharibans et al., 2017). EGG can reflect the existence of gastric motility disorders in many diseases, such as diabetes, gastroparesis, functional dyspepsia, gastroesophageal reflux disease and motion sickness, etc (Koch, 2001; Sha et al., 2009; Yin and Chen, 2013; Zhang et al., 2016; Al Kafee and Akan, 2018). Lang et al. found that in 70% of subjects, motion sickness is accompanied by a decrease in gastrointestinal motility, and EGG can be used to evaluate gastric motility during this period (Lang et al., 1999; Zhang et al., 2016). Van Dyck et al. recorded gastric myoelectric activity in patients with overeating through EGG, and found that the response to satiety in overeating was delayed (van Dyck et al., 2020). In terms of treatment, Zhang et al. found that the main power of octreotide in the treatment of esophageal variceal bleeding was significantly reduced, and the change of main power could evaluate the efficacy of octreotide (Zhang et al., 2013). In addition, some studies have used EGG parameters to compare the changes of gastric motility before and after the treatment of gastric motility drugs in patients with sepsis (Mancilla Asencio et al., 2019). However, there is no research showing the application of EGG in AP. Therefore, the purpose of this study was to reflect the presence of gastric motility disorders in AP patients through EGG, and to explore the value of EGG-related parameters in predicting the severity of AP.

## 2 Materials and methods

### 2.1 Study design and participants

According to the Atlanta classification of acute pancreatitis revised in 2012 (Banks et al., 2013), Patients diagnosed with AP in the First Affiliated Hospital of Nanchang University from June 2020 to December 2020 were included. The exclusion criteria of patients including: 1) Admission >72 h after the onset of AP; 2) less than 18 years old or more than 80 years old; 3) patients with a history of diabetes, cirrhosis, gastric cancer or other gastrointestinal motility disorders; 4) Previous subtotal gastrectomy/total gastrectomy or other abdominal surgery; 5) Patients who took gastrointestinal motility drugs within 3 days before the onset of the disease. At the same time, 12 healthy adults were selected as control group. The Ethics Committee of the First Affiliated Hospital of Nanchang University approved the study (No. 2019099).

### 2.2 Data collection and measurement

**FIGURE 1:**
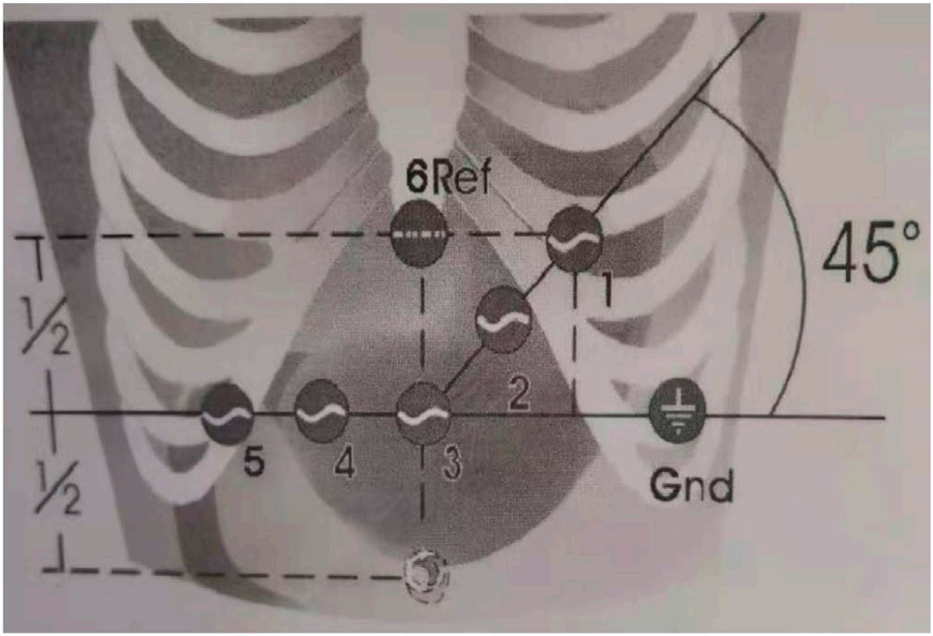
EGG electrode placement. Lead I-III: stomach; Lead IV: pylorus; Lead V: duodenum; Lead VI: reference electrode.

Five EGG parameters (Percentage of normal gastric slow wave (PNGSW), main frequency, average frequency, percentage of gastric tachycardia (PGT), percentage of gastric bradycardia (PGB)) were collected in this study. EGG examination process: 1) Patient position: take the supine position, keep quiet and awake during the examination, avoid talking, calling and falling asleep; 2) Skin preparation: thoroughly clean the abdominal skin where the electrode is placed. If the body hair is too much, it needs to be shaved, and the conductive paste is applied to reduce the skin impedance. Place the electrode as shown in Figure 1 Lead VI: under the xiphoid process; lead III: the midpoint of the umbilical and xiphoid connection; lead I: make a horizontal line with lead III, lead I is located on the left side of lead VI and is 45° to the horizontal line; lead II: located at the midpoint of the connection between leads I and III; lead V: the intersection of the level of lead III and the rib arch; lead IV: located at the midpoint of the connection between lead III and lead V; lead VI: lead III on the left side of about 10 cm; 3) Recording time: EGG was recorded on an empty stomach for at least 30 min (Yin and Chen, 2013). Spectrum analysis based on fast Fourier transform (Komorowski and Pietraszek, 2016), EGG software was used to obtain the Five EGG parameters (PNGSW, main frequency, average frequency, PGT, PGB). The breathing belt is tied to the chest, and the tightness is appropriate to be close to the chest wall but does not affect the patient’s breathing. The artifacts caused by duodenum and respiration were removed during the analysis.

Refer to the published research and the Dutch MMS gastrointestinal motility examination system used in this study (Lim et al., 2012; Yin and Chen, 2013; Popović et al., 2019), The definition is as follows: 1). Normal gastric slow wave frequency 2.4-3.6 times/min (CPM); Bradygastria <2.4 CPM; Tachygastria >3.6 CPM; 2) The PNGSW: the percentage of time when the frequency of gastric slow wave is in the range of 2.4–3.6 CPM, normal ≥70%; the PGB and PGT was the percentage of time within the frequency <2.4 CPM and >3.6 CPM respectively.

In addition, the following information was also collected: 1) Demographic characteristics, including age, gender, AP etiology, smoking and drinking history, hypertension, etc.,; 2) Laboratory examination and severity score (collected within 24 h of admission), including serum albumin level, C-reactive protein level (CRP), procalcitonin level, D-dimer level, urea nitrogen level, blood glucose level, calcium level, interleukin 6 (IL-6), interleukin 1β (IL-1β), acute physiology and chronic health evaluation (APACHE) II score, bedside index for severity in AP (BISAP score), modified CT severity index score (mCTSI score) and systemic inflammatory response syndrome (SIRS score); 3) Clinical outcomes included length of hospital stay and total cost of hospitalization.

### 2.3 Statistical analyses

The measurement data of normal distribution were expressed as mean ± standard deviation, using *t*-test or variance analysis. The measurement data of non-normal distribution were expressed as median (interquartile range), using non-parametric test. Count data were expressed as frequency (percentage), using chi-square test or Fisher exact test. The receiver operating characteristic curve (ROC curve) was constructed, and the predictive value of EGG parameters on the severity of AP was evaluated by calculating the area under the ROC curve. *p* < 0.05 was considered statistically significant. Data were analyzed using SPSS software (v26.0).

## 3 Results

### 3.1 Patients with AP have gastric motility disorders

A total of 83 AP patients were included in this study. According to the Atlanta classification criteria, there were 41 patients with MAP, 36 patients with MSAP, and 6 patients with SAP. The average age was 46.55 ± 13.36 years old, including 57 males (69.4%). The main causes of AP were gallstones (33.7%), hypertriglyceridemia (37.3%), alcohol consumption (4.8%), post-ERCP (2.4%) and mixed (9.6%). Another 10 cases (12.0%) were diagnosed as idiopathic AP due to unknown etiology. Most studies have shown that AP patients have gastrointestinal motility disorders. In order to objectively demonstrate this fact, 12 healthy normal people were selected as the control group, and EGG examination was performed on them and AP patients. As shown in Table 1, there was no significant difference in age and gender between the two groups. The PNGSW in AP patients was significantly lower than that in the control group (*p* < 0.05), while the PGB was significantly higher than that in the control group (*p* < 0.05). However, there were no significant differences in the mean frequency, the main frequency and the percentage of gastric tachycardia between the two groups (*p* > 0.05), which may be related to the majority of mild patients in the case group.

**TABLE 1:** Social characteristics and EGG parameters of AP patients and normal healthy people.

Variables	Control group (*n* = 12)	AP group (*n* = 83)	*p*-Value
Age (years)	46.1 ± 14.1	46.6 ± 13.4	0.910^a^
Sex (male)	9 (75%)	57 (68.7%)	0.657^b^
Average frequency	2.9 ± 0.2	2.8 ± 0.2	0.208^a^
Main frequency	2.8 (2.2–3.1)	2.6 (2.1–3.1)	0.623^c^
PNGSW(%)	82.3 ± 16.1	68.7 ± 18.7	0.021^a^
PGB (%)	13.6 ± 12.7	28.1 ± 17.8	0.008^a^
PGT (%)	0 (0–3.3)	0 (0–3.9)	0.914^c^

AP, acute pancreatitis; PNGSW, percentage of normal gastric slow wave; PGT, percentage of gastric tachycardia; PGB, percentage of gastric bradycardia; “a”, *t*-test; “b”, Fisher exact test; “c”, non-parametric test.

### 3.2 The degree of gastric motility disorder reflected by EGG is related to the severity of AP

**FIGURE 2:**
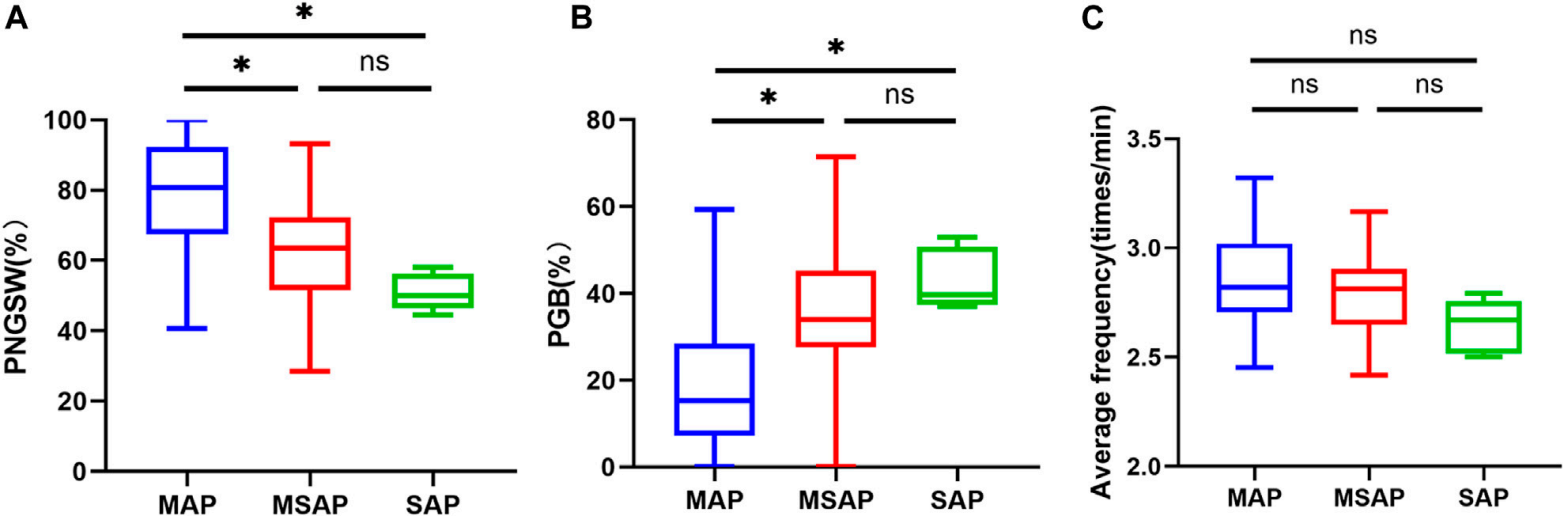
EGG parameters **(A)**: PNCSW; **(B)** PGB; **(C)** average frequency) of MAP, MSAP and SAP were compared among groups. PNGSW, percentage of normal gastric slow wave; PGB, percentage of gastric bradycardia. “*”, *p* < 0.05.

After clarifying the existence of gastric motility disorders in AP patients, this study intends to further explore whether EGG-related parameters are related to the severity of AP. As shown in Table 2, inflammatory markers (CRP, procalcitonin, IL-6) and various AP severity-related system scores (mCTSI score, APACHE II score, BISAP score, SIRS score) were significantly different among MAP, MSAP and SAP groups. In addition, with the increase of the severity of the disease, the number of days of hospitalization and the total cost of hospitalization also increased. By comparing the EGG parameters among the three groups, we found that the average frequency, the PNGSW and the PGB were statistically significant among the three groups (*p* < 0.05), while the main frequency and the PGT were not significantly different among the groups (Table 2). Subsequently, the Bonferroni method was used for pairwise comparison and analysis. It was found that the PNGSW and the PGB were only significantly different between MAP and MSAP, MAP and SAP (*p* < 0.05), while the average frequency was not significantly different between any two groups (Figure 2).

**TABLE 2:** Characteristics of the acute pancreatitis patients.

Variables	MAP (*n* = 41)	MSAP (*n* = 36)	SAP (n = 6)	*p*-Value
Sex (male)	23 (56.1%)	29 (80.6%)	5 (83.3%)	0.045^b^
Age (years)	47.9 ± 13.8	44.2 ± 12.2	51.7 ± 16.2	0.310^a^
Smoking	12 (29.3%)	12 (33.3%)	3 (50%)	0.585^b^
Alcohol	13 (31.7%)	11 (30.6%)	3 (50%)	0.627^b^
Hypertension	6 (14.6%)	4 (11.1%)	2 (33.3%)	0.283^b^
Etiology				0.042^b^
Biliary	18 (43.9%)	10 (27.8%)	0 (0.0%)	
Hypertriglyceridemia	14 (34.1%)	13 (36.1%)	4 (66.7%)	
Alcoholic	4 (9.8%)	0 (0.0%)	0 (0.0%)	
Idiopathic	3 (7.3%)	6 (16.7%)	1 (16.7%)	
Others	2 (4.9%)	7 (19.4%)	1 (16.7%)	
Glucose (mmol/l)	6.8 (5–8.3)	6.6 (5.4–8.7)	6.3 (5.7–8.3)	0.132^c^
Albumin (g/dl)	42.3 (38.3–46.0)	42 (35.9–45.7)	36.8 (36–38.8)	0.142^c^
CRP (mg/dl)	57.7 (13.6–102.3)	158.5 (86.9–223.5)	212.0 (198.8–417)	0.000^c^
Procalcitonin (ng/mL)	0.3 (0.3–0.3)	0.5 (0.3–1.7)	4.9 (1–7.1)	0.000^c^
IL-6 (ng/l)	32.7 (12.0–109.7)	116.1 (42.4–330.4)	149.3 (57.9–358.3)	0.021^c^
IL-1β(ng/l)	5.4 (4.7–24.9)	21.2 (10.7–30.2)	14.0 (2.1–29.8)	0.662^c^
D-dimer (mg/l)	0.6 (0.3–1.7)	1.3 (0.6–2.1)	1.7 (0.8–4.7)	0.109^c^
Creatinine (umol/l)	64.5 (56.2–73.2)	62.6 (47.4–76.0)	78.1 (60.0–106.1)	0.191^c^
Urea nitrogen (mg/dl)	4.3 (2.7–5.5)	4.7 (3.0–6.5)	5.9 (3.8–9.3)	0.297^c^
Calcium (mmol/l)	2.2 ± 0.2	2.2 ± 0.2	2.1 ± 0.2	0.372^a^
mCTSI score	2 (2–2)	6 (4–8)	8 (6–8.5)	0.000^c^
BISAP score	1 (0–1)	1 (1–2)	2 (1.75–3)	0.001^c^
APACHE II score	2 (1–4)	3 (2–5)	7.5 (5.5–10.5)	0.001^c^
SIRS score	0 (0–1)	1 (0–1.75)	2 (1.75–3)	0.002^c^
Hospital days	7 (5–8.5)	10 (8–13.8)	13.5 (7.8–19.3)	0.000^c^
Hospitalization expenses (yuan)	13,634.8 (11,516.5–26,875)	23,847.7 (15,672.6–44,510.7)	32,179.6 (14,045.5–69,625.6)	0.001^c^
Mean frequency	2.9 ± 0.2	2.8 ± 0.2	2.6 ± 0.1	0.035^a^
Main frequency	2.6 (2.1–3.2)	2.7 (2.1–3.1)	2.2 (2.1–2.4)	0.180^c^
PNGSW(%)	78 ± 16.8	61.7 ± 16.8	50.9 ± 5.1	0.000^a^
PGB (%)	15.4 (7.3–28.5)	34.0 (27.7–45.3)	39.7 (37.4–50.7)	0.000^c^
PGT (%)	0 (0–3.7)	0 (0.0–5.6)	5.3 (0–13.1)	0.228^c^

AP, acute pancreatitis; PNGSW, percentage of normal gastric slow wave; PGT, percentage of gastric tachycardia; PGB, percentage of gastric bradycardia. “a”, *t*-test; “b”, Fisher exact test; “c”, non-parametric test.

### 3.3 The value of EGG parameters and other indicators in predicting the severity of AP

**FIGURE 3:**
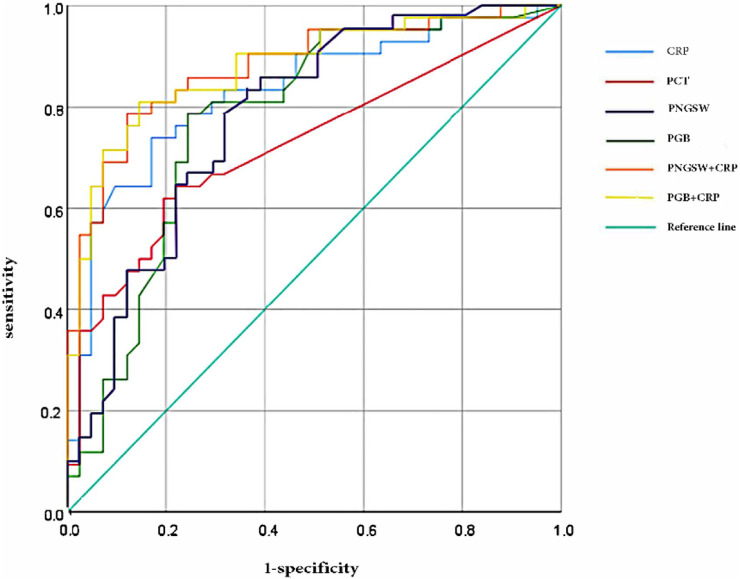
The ROC curve of N-MAP was predicted by CRP, PCT, PNGSW, PGB, PNGSW + CRP and PGB + CRP. PNGSW, percentage of normal gastric slow wave; PGB, percentage of gastric bradycardia.

According to the above results, AP patients were divided into two groups: mild acute pancreatitis group (MAP) and non-mild acute pancreatitis group (N-MAP, including MASP and SAP). ROC curve was constructed, and the value of each index in predicting N-MAP was evaluated by calculating the area under the ROC curve (AUC) (Figure 3; Table 3). Among them, CRP had the highest accuracy in predicting N-MAP (AUC = 0.83), but its sensitivity was slightly lower than that of slow wave percentage and bradygastria percentage. The AUC of PNGSW and PGB in predicting N-MAP were 0.777 (95% CI 0.676-0.877, *p* < 0.001) and 0.775 (95% CI 0.67-0.879, *p* < 0.001), respectively. The cutoff values of N-MAP PNGSW and PGB calculated by Youden index were 72.21% (sensitivity 78.6%, specificity 68.3%, positive likelihood ratio 2.479, negative likelihood ratio 0.313) and 27.89% (sensitivity 78.6%, specificity 75.6%, positive likelihood ratio 3.221, negative likelihood ratio 0.283), respectively. In general, the accuracy of PNGSW and PGB in predicting N-MAP was similar, but the specificity of PGB was higher. Then the ROC curve was constructed with the combination of PNGSW + CRP and PGB + CRP (Figure 3; Table 3). The accuracy of the two combinations in predicting N-MAP was improved. The PNGSW + CRP only increased the specificity by 4.9%, while the sensitivity and specificity of the PGB + CRP combination increased.

**TABLE 3:** The value of percentage of CRP, PCT, PNGSW, PGB, PNGSW + CRP and PGB + CRP in predicting N-MAP.

	Youden index	Sensitivity	Specificity	AUC	*p*-Value
PNGSW	0.468	78.6	68.3	0.777	0.000
PGB	0.542	78.6	75.6	0.775	0.000
CRP	0.567	73.8	82.9	0.830	0.000
PCT	0.424	61.9	80.5	0.726	0.000
PNGSW + CRP	0.664	78.6	87.8	0.880	0.000
PGB + CRP	0.663	81.0	85.4	0.878	0.000

AUC, the area under the ROC, curve. The Youden index (sensitivity + specificity-1), The greater the index, the better the effect of the screening experiment and the greater the authenticity.

## 4 Discussion

EGG is an examination that monitors the myoelectric activity of gastric smooth muscle by placing electrodes in the abdomen. When it is correctly recorded, EGG-related parameters can reflect gastric motility. Normal gastric myoelectric activity includes slow wave and spike (action potential). Slow wave determines the maximum frequency and propagation direction of gastric contraction. When gastric slow wave is accompanied by spike, gastric contraction occurs. It is worth noting that not every gastric contraction is accompanied by spike (Yin and Chen, 2013; Wolpert et al., 2020). Studies have shown that there is a good correlation between the frequency of gastric slow wave detected by serous EGG and abdominal surface EGG, and the main frequency of EGG can accurately represent the frequency of gastric slow wave, which lays a certain foundation for the clinical development of EGG (Chang, 2005; Yin and Chen, 2013). A large number of studies have shown the role of EGG in the diagnosis of clinical disease-related gastric motility disorders (Koch, 2001; Sha et al., 2009; Yin and Chen, 2013; Zhang et al., 2016; Al Kafee and Akan, 2018). AP patients usually have gastrointestinal motility disorders, but there is no research on EGG and AP.

To the best of our knowledge, this observational, cohort study is the first to link EGG and AP. Through strict and standardized EGG examination of healthy normal people and AP patients, and based on fast Fourier transform spectrum analysis, it was found that the percentage of normal gastric slow wave in AP patients was significantly lower than that in control group, while the percentage of bradygastria was significantly higher than that in control group, which objectively proved the existence of gastric motility disorder in AP patients. It may be because the sample size included in this study is small, and the proportion of mild symptoms in AP patients is relatively high, so there is no statistical difference in the main frequency and average frequency. On this basis, AP patients were divided into MAP, MSAP and SAP groups. It was found that there were significant differences in inflammatory factors CRP, PCT and various AP severity scoring systems among the three groups. Similarly, EGG-related parameters, such as mean frequency, percentage of normal gastric slow waves, and percentage of bradygastria, were also statistically significant in AP with different severity. Unfortunately, we found that there were significant differences in the percentage of normal gastric slow waves and the percentage of bradycardia between MAP and MSAP, MAP and SAP, but there was no significant difference in the average frequency between any two groups.

Therefore, the patients were further divided into MAP and N-MAP groups. By constructing the ROC curve, it was found that some parameters of EGG, the percentage of normal gastric slow wave (cut-off value of 72.21%) and the percentage of bradygastria (cut-off value of 72.21%) could be used to predict the occurrence of N-MAP, with a sensitivity of 78.6%, slightly higher than that of CRP (73.8%). The combination of EGG parameters and CRP showed that the diagnostic performance was improved. The percentage of bradycardia combined with CRP to predict N-MAP could improve the sensitivity and maintain high specificity. In this study, the sensitivity of CRP to predict the occurrence of N-MAP was only 73.8%, which was much lower than 88% of Sternby et al. (Sternby et al., 2017), This may be related to the small sample size, but the sensitivity and specificity were improved after combining the percentage of gastric bradycardia.

There are still some limitations in this study, Firstly, the sample size of this study is too small, especially SAP; Secondly, this study did not prove that there was a statistical difference in EGG parameters between MSAP and SAP, which may be related to the small sample size; Thirdly, because AP is in the early stage of fasting, the examination in this study belongs to the improved EGG, and EGG parameters of at least 30 min are recorded only in the fasting state. Therefore, there is no statistical analysis of the main power, and the postprandial/preprandial power ratio cannot be calculated. However, we believe that this has little effect on the results of the study, because the absolute value of the main power of EGG has no physiological and clinical significance (Liu et al., 2014). Fourthly, although we have proved that there is a correlation between the percentage of normal gastric slow wave, the percentage of bradygastria and CRP, the correlation is not close and the causal relationship is not proved. Therefore, further research is needed.

## 5 Conclusion

In conclusion, this study proves that EGG is a new, non-invasive and simple method in AP, objective examination of gastric motility can be applied to AP patients in the early stage of clinical practice to predict the occurrence of N-MAP, which is conducive to timely intervention to prevent the aggravation of AP. However, the results of this study still need to be further verified by a large number of multi-center, large sample size prospective studies, and dynamically observe the changes of EGG parameters during the course of disease.

## Data Availability

The original contributions presented in the study are included in the article/Supplementary material, further inquiries can be directed to the corresponding author.
